# Calicivirus RNA-Dependent RNA Polymerases: Evolution, Structure, Protein Dynamics, and Function

**DOI:** 10.3389/fmicb.2019.01280

**Published:** 2019-06-06

**Authors:** Elena Smertina, Nadya Urakova, Tanja Strive, Michael Frese

**Affiliations:** ^1^Commonwealth Scientific and Industrial Research Organisation, Health and Biosecurity, Canberra, ACT, Australia; ^2^Faculty of Science and Technology, University of Canberra, Canberra, ACT, Australia; ^3^Department of Entomology, Pennsylvania State University, University Park, PA, United States; ^4^Invasive Animals Cooperative Research Centre, University of Canberra, Canberra, ACT, Australia

**Keywords:** polymerase, RdRp, replication, motif, RNA virus, *Caliciviridae*, *Lagovirus*, RHDV

## Abstract

The *Caliciviridae* are viruses with a positive-sense, single-stranded RNA genome that is packaged into an icosahedral, environmentally stable protein capsid. The family contains five genera (*Norovirus*, *Nebovirus, Sapovirus*, *Lagovirus*, and *Vesivirus*) that infect vertebrates including amphibians, reptiles, birds, and mammals. The RNA-dependent RNA polymerase (RdRp) replicates the genome of RNA viruses and can speed up evolution due to its error-prone nature. Studying calicivirus RdRps in the context of genuine virus replication is often hampered by a lack of suitable model systems. Enteric caliciviruses and RHDV in particular are notoriously difficult to propagate in cell culture; therefore, molecular studies of replication mechanisms are challenging. Nevertheless, research on recombinant proteins has revealed several unexpected characteristics of calicivirus RdRps. For example, the RdRps of RHDV and related lagoviruses possess the ability to expose a hydrophobic motif, to rearrange Golgi membranes, and to copy RNA at unusually high temperatures. This review is focused on the structural dynamics, biochemical properties, kinetics, and putative interaction partners of these RdRps. In addition, we discuss the possible existence of a conserved but as yet undescribed structural element that is shared amongst the RdRps of all caliciviruses.

## Introduction

**FIGURE 1:**
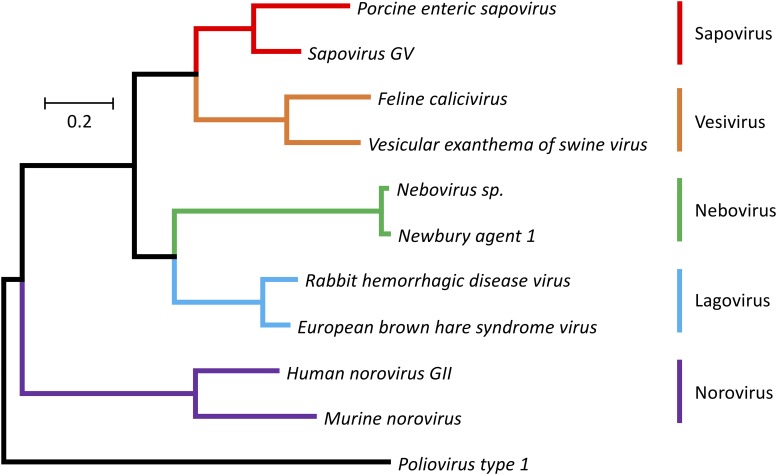
Phylogenetic tree for RdRp protein sequences of the family *Caliciviridae* and *Poliovirus type 1* (Mahoney strain). The evolutionary history was inferred using the Maximum Likelihood method (Jones et al., 1992). The tree is drawn to scale, with branch lengths representing the number of substitutions per site. The analysis involved amino acid sequences from 11 caliciviruses [*Porcine enteric sapovirus*, A0A348BR93 (UniProt); *Sapovirus GV*, NP783310 (NCBI Protein); *Feline calicivirus*, NP786896 (NCBI Protein); *Vesicular exanthema of swine virus*, AYN44917 (NCBI Protein); *Nebovirus sp*., YP529897 (NCBI Protein); *Newbury agent 1*, NP740332 (NCBI Protein); *Rabbit haemorrhagic disease virus*, NP786902 (NCBI Protein); *European brown hare syndrome virus*, D0UGI3 (UniProt); *Human norovirus GII*, AWB14625 (NCBI Protein); *Murine norovirus*, P03300 (UniProt)] and a poliovirus [*Poliovirus type 1*, Q6IX02 (UniProt)]. Evolutionary analyses were conducted using the MEGA7 program package (Kumar et al., 2016). Different colors are used for different calicivirus genera.

The *Caliciviridae* family currently consists of five genera (*Norovirus*, *Nebovirus, Sapovirus*, *Lagovirus*, and *Vesivirus*) (Figure 1). The establishment of three additional genera (i.e., *Recovirus*, *Valovirus*, and *Balovirus*) has been proposed (Farkas et al., 2008; L’Homme et al., 2009; Wang et al., 2017). The exponential increase in metagenomic sequence data is likely to reveal an even higher degree of diversity for this virus family. Not surprisingly, many of the currently known caliciviruses are highly pathogenic (a characteristic that usually leads to discovery), but research using metagenomics is likely to discover more non-pathogenic family members (Mahar et al., 2019). Viruses from the genera *Norovirus* and *Sapovirus* are a common cause of gastroenteritis in humans and animals. For example, *Norwalk virus* and other human noroviruses are responsible for almost half of all gastroenteritis cases globally (Atmar and Estes, 2006). These viruses are easily transmitted either directly from person to person or through contaminated food and water. Infections are especially dangerous for elderly, young, and immunocompromised individuals such as transplant recipients (Schwartz et al., 2011). Despite the high socioeconomic costs associated with human norovirus outbreaks, no approved vaccines or small molecule inhibitors are currently available to prevent or cure infections. Viruses from the genus *Vesivirus*, such as *Feline calicivirus* (FCV) and *Vesicular exanthema of swine virus* (VESV), are highly contagious in animals and can cause persistent infections. FCV causes fever and acute upper respiratory tract and oral cavity disease in all feline species and can lead to a virulent systemic disorder (Hurley and Sykes, 2003). VESV affects pigs and marine mammals, causing fever and epithelial lesions around the mouth, nostrils, and on the feet (Neill et al., 1995). The genus *Lagovirus* comprises only viruses that infect lagomorphs, especially rabbits and hares. Pathogenicity among lagoviruses can differ dramatically. The *Rabbit haemorrhagic disease virus* (RHDV) causes acute necrotizing hepatitis and disseminated intravascular coagulation in European rabbits (*Oryctolagus cuniculus*), which leads to death 48–72 h post-infection, while the *Rabbit calicivirus* (RCV) causes only mild disease manifestations (Abrantes et al., 2012). Since the mid-1990s, RHDV has been used to control rabbit populations in Australia and New Zealand following the introduction of the European rabbit in the late 1800s (Cooke, 2002; Cooke and Fenner, 2002). Even though RHDV is an important biocontrol agent, it has not yet been studied in great detail; many aspects of viral replication and the function of several proteins remain unknown.

**FIGURE 2:**
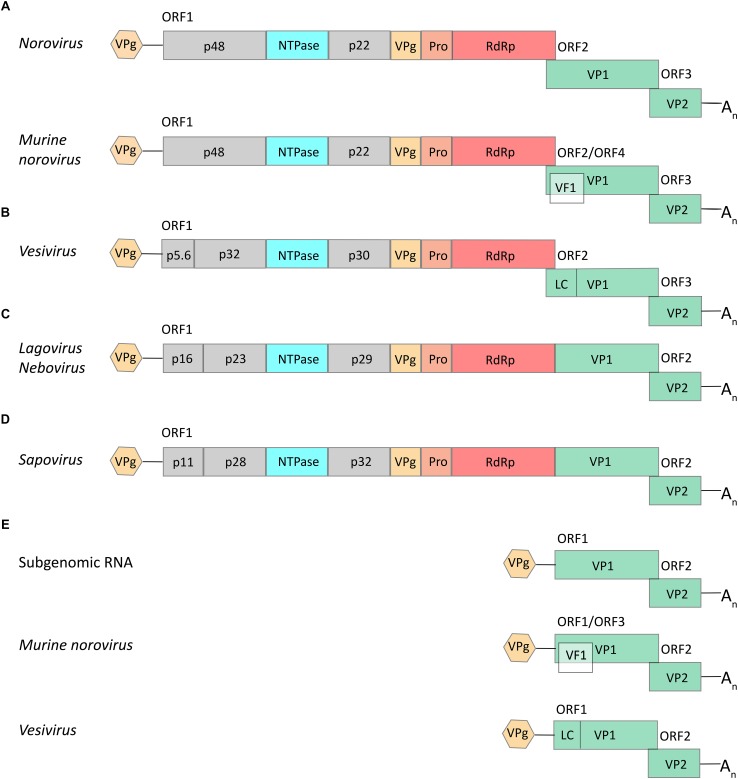
Schematic representations of typical calicivirus genome organizations. **(A–D)** Genomic full-length RNAs of about 7.5 kb in size contain either two ORFs (in viruses of the genera *Lagovirus*, *Nebovirus*, and *Sapovirus*) or three ORFs (*Norovirus* and *Vesivirus*), except for the genomic RNA of *Murine norovirus* (MNV; genus *Norovirus*) that may contain an additional ORF (encoding the VF1 protein). **(E)** All caliciviruses except MNV and vesiviruses have subgenomic RNAs of about 2.1 kb in size with two ORFs that encode the main structural proteins, VP1 and VP2; the subgenomic RNA of MNV includes three ORFs (similar to the corresponding genomic RNA) and the subgenomic RNA of vesiviruses encodes – apart from proteins VP1 and VP2 – a small leader of the capsid protein (LC). Colored boxes represent coding sequences that are flanked by untranslated leader and trailer sequences (shown as lines). Hexagons represent VPg proteins that are covalently bound to the 5′ end of all genomic and subgenomic RNAs; A_n_ represents the poly(A) tail at the 3′ end of all genomic and subgenomic RNAs.

Viruses of the *Caliciviridae* family share a number of features. The genome consists of positive-sense, single-stranded RNAs that contain coding sequences in two or more partially overlapping open reading frames (ORFs). The coding sequences are flanked by untranslated regions (UTRs) at both the 5′ and 3′ ends. Genomic RNAs are covalently linked at the 5′ end to a viral protein (VPg, for “virion protein, genome-linked”) and are polyadenylated at the 3′ end. Calicivirus particles contain two types of RNA, a genomic (full-length) RNA of about 7.5 kb and one or more copies of a subgenomic RNA of about 2 kb (Ehresmann and Schaffer, 1977; Meyers et al., 1991a,b). The number of ORFs varies from two to four in full-length genomic RNAs and from two to three in subgenomic RNAs (Wirblich et al., 1996; McFadden et al., 2011; Figure 2). ORF1 is always the largest of the reading frames and encodes a polyprotein that is subsequently cleaved into five non-structural proteins and VPg (genus *Norovirus* and *Vesivirus*) or five non-structural proteins, VPg, and the major capsid protein VP1 (genus *Lagovirus*, *Nebovirus*, and *Sapovirus*) (Martín Alonso et al., 1996; Meyers et al., 2000). The second and third ORFs in the genomic RNA of noroviruses encode the structural proteins VP1 and VP2, respectively. In vesiviruses, ORF2 encodes the VP1 precursor protein that is subsequently cleaved into a mature VP1 and a small leader peptide (leader of the capsid protein, LC). The LC protein of FCV is cytopathic and promotes virus spread (Abente et al., 2013). The subgenomic RNAs of all genera are very similar to each other; they contain the 5′ UTR and the VP1 and VP2 coding sequences (Meyers et al., 1991a,b, 2000; Boga et al., 1992). In *Murine norovirus* (MNV), there is an additional ORF in the VP1 coding region of both genomic and subgenomic RNAs that encodes the viral factor 1 (VF1), an antagonist of the innate antiviral immune response (McFadden et al., 2011).

The structural protein VP1 forms an icosahedral, non-enveloped capsid of about 25–40 nm in diameter (Parra and Prieto, 1990; Prasad et al., 1994, 1999). A typical calicivirus capsid contains 90 VP1 dimers. Protruding VP1 (VP60 in RHDV) domains create a surface topography that resembles cup-shaped depressions when viewed using electron microscopy, which inspired the name “calicivirus” (Latin “*calyx*” = cup). The basic VP2 protein has also been found associated with virus particles (although in much smaller numbers) and plays a role in RNA replication and the maturation of infectious virus particles (Sosnovtsev et al., 2005). In addition, recent studies of FCV suggest a role for VP2 in the formation of a portal-like structure facilitating the delivery of viral RNA into the cytoplasm in the early stages of infection (Conley et al., 2019).

The VPg protein is also found in virus particles and should therefore be categorized as a structural protein, since the components of a mature virus particle are defined as structural proteins. The VPg is covalently linked to the 5′ end of both the full-length genomic and subgenomic RNAs (Black et al., 1978; Burroughs and Brown, 1978; Meyers et al., 1991a). Mass-spectrometry-based assays showed that FCV and MNV VPg proteins are linked to a guanosine diphosphate moiety via tyrosine residues, which is consistent with the presence of a highly conserved 5′ guanosine nucleotide in the genome of all caliciviruses (Olspert et al., 2016). The association between VPg and RNA was recognized for the first time when, following phenol extraction, a significant amount of caliciviral RNA was found in the interphase, along with other viral and cellular proteins. However, when the samples were treated with protease K prior to the extraction, the viral RNA was found in the aqueous phase. Furthermore, when purified RHDV RNA was labeled with ^125^I, autoradiography revealed two protein bands corresponding to genomic and subgenomic RNAs. The subsequent treatment of the labeled RNAs with RNase produced a single band of about 15 kDa on SDS-PAGE (sodium dodecyl sulfate-polyacrylamide gel electrophoresis) (Meyers et al., 1991a). The VPg protein also plays a critical role in RNA replication. Following nucleotidylation by the RNA-dependent RNA polymerase (RdRp) or an RdRp precursor, VPg can act as a primer for genome replication (Belliot et al., 2008; Goodfellow, 2011).

The non-structural proteins can be categorized into those with known functions (NTPase, 3C-like protease, and RdRp) and unknown functions (all remaining proteins). The first one or two N-terminal proteins of the full-length genomic RNA (e.g., p16, p23, and p29 in lagoviruses, or p48 and p22 in noroviruses) may have functions similar to the so-called “security proteins” of the *Picornaviridae* family that counteract host defense mechanisms (Agol and Gmyl, 2010). This hypothesis is based on the fact that the coding sequence for the calicivirus proteins and the picornavirus security proteins have a similar position in the genome of the respective viruses. Although the calicivirus proteins do not share detectable sequence homologies with their picornavirus counterparts, accumulating data from functional studies suggest that these proteins do indeed impede immune responses, e.g., those that depend on cellular secretory pathways. The Norwalk virus protein p48 (when expressed as a recombinant protein in transfected cells) induces Golgi membrane rearrangements (Fernandez-Vega et al., 2004). The p48 protein of both MNV and human noroviruses interacts with the vesicle-associated membrane protein-associated protein A (VAP-A). VAP-A is a soluble *N*-ethylmaleimide-sensitive factor attachment protein receptor (SNARE)-regulator and is involved in vesicle transport (Weir et al., 1998; Ettayebi and Hardy, 2003). This interaction is likely to disrupt intracellular protein trafficking, as cells that express p48 were unable to expose the vesicular stomatitis G glycoprotein on the cell surface (Ettayebi and Hardy, 2003). Moreover, strand-specific quantitative PCR revealed a delayed accumulation of positive and negative strand MNV RNAs in VAP-A deficient cells (McCune et al., 2017). The p22 protein of Norwalk virus also contributes to Golgi disaggregation and blocks trafficking of vesicles from the ER to the Golgi (Sharp et al., 2010). However, the corresponding proteins in other calicivirus genera have not yet been functionally characterized and, to date, no conserved motifs have been identified that would suggest particular functions. Therefore, their exact role in virus replication and/or pathogenesis remains unknown.

The functions of the remaining non-structural proteins were deduced by comparing calicivirus and picornavirus sequences and by searching for conserved motifs. A 2C-like helicase (named NTPase in Figure 2) was identified after the detection of a nucleotide-binding site that is typical for viral proteins (Neill, 1990). Later, this enzyme was shown to be associated with the replication complex and to destabilize double-stranded RNA in an NTP-independent manner, representing an unexpected RNA chaperone-like activity (Li et al., 2017; Han et al., 2018). Thereafter, the p58 cleavage product of the RHDV polyprotein was found to resemble the 3D polymerase of poliovirus, and its role in RNA replication was subsequently confirmed using functional assays (Wirblich et al., 1996; Vazquez et al., 1998). Similarly, the sequence preceding the RdRp gene was suggested to code for a 3C-like protease (Neill, 1990; Jiang et al., 1993). As with the picornavirus proteases, the calicivirus homologs are responsible for the processing of the polyprotein (on a par with cellular proteases) and for the formation and accumulation of a 3CD-like polymerase precursor (Sosnovtseva et al., 1999; Thumfart and Meyers, 2002; Oka et al., 2005).

RNA-dependent RNA polymerases are the key proteins responsible for viral replication. In all caliciviruses, the RdRp coding sequence follows that of the viral protease at the 3′end of ORF1. Mature RdRps are proteins of about 60 kDa (75 kDa in the precursor form). Remarkably, the calicivirus RdRp precursor protein is also an active polymerase enzyme (Wei et al., 2001). RdRps are usually among the best-characterized proteins of any given virus species; RdRps from several caliciviruses have been crystallized and studied (Table 1).

**Table 1:** Polymerase crystal structures and amino acid sequence information for representative members of the *Caliciviridae* family.

Genus	Species	PDB code	UniProt entry	References
*Norovirus*	*Norwalk virus*	1SH0	Q83883	Ng et al., 2004
	*Murine norovirus* (MNV)	3NAH	Q80J95	Lee et al., 2011
*Vesivirus*	*Feline calicivirus* (FCV)	No data	Q66914	
	*Vesicular exanthema of swine virus* (VESV)	No data	Q9DUN3	
*Sapovirus*	*Sapporo virus*	2CKW	Q69014	Fullerton et al., 2007
*Lagovirus*	*Rabbit haemorrhagic disease virus* (RHDV)	1KHW	P27411	Ng et al., 2002
	*Rabbit calicivirus* (RCV)	No data	A0A1B2RX11	


## Features Common to All Calicivirus RdRps

**FIGURE 3:**
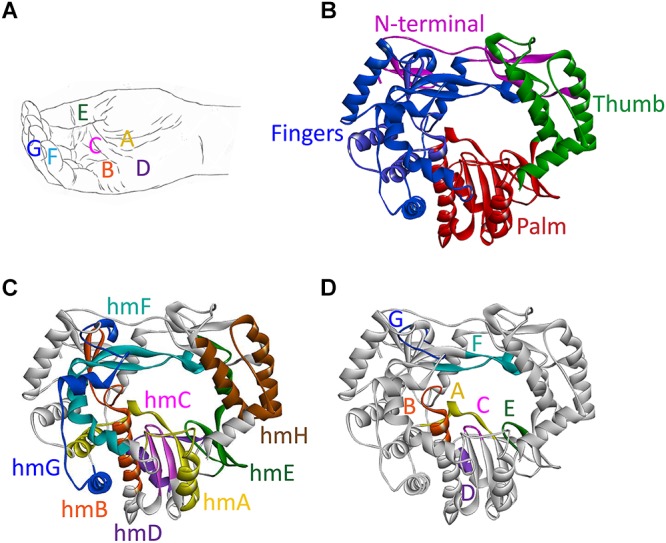
Domains, motifs, and homomorphs of a typical calicivirus RdRp. **(A)** Representation of a slightly cupped right hand resembling an RdRp with the position of motifs A to G on fingers, palm, and thumb. **(B–D)** Ribbon diagrams of the RHDV RdRp (PDB ID: 1KHW); **(B)** fingers, palm, and thumb domains colored blue, red, and green, respectively, and the N-terminal domain colored magenta; **(C)** structurally conserved homomorphs (hmA to hmH); and **(D)** functional motifs A to G (the positions of homomorphs and corresponding motifs are indicated by the same color). Ribbon diagrams were generated using Discovery Studio (Dassault Systèmes BIOVIA, Discovery Studio Visualizer v17.2.0, San Diego: Dassault Systèmes, 2016).

The shape of all RdRps resembles a right hand with fingers, palm, thumb, and an N-terminal domain that links the finger and thumb domains (Figure 3A,B). The active site of the enzyme is located in the palm domain and its architecture is highly conserved. So far, seven highly conserved amino acid sequence motifs have been identified: four motifs in the palm domain (motifs A, B, C, and D), one motif in the thumb domain (motif E), and two motifs in the fingers domain (motifs F and G) (Figure 3A,D; Poch et al., 1989; Koonin, 1991). Whereas these short functional motifs have highly conserved amino acid sequences, the so-called homomorphs encompassing these motifs [except for the newly discovered homomorph H (Černý et al., 2014)] represent protein regions with a conserved structure but no recognizable consensus sequence (Lang et al., 2013; Figure 3C).

Individual motifs cooperate to perform highly specialized functions. Motifs B, D, E, and F are involved in nucleotide recognition and coordination, motifs B and G coordinate template and primer binding, and motifs A and C execute the catalysis of nucleotide binding (Ng et al., 2008; Choi, 2012; Table 2). Motif A comprises two Asp residues separated by up to five amino acids, whereas motif C includes an Asp-Asp dipeptide, forming the highly conserved Gly-Asp-Asp motif (Poch et al., 1989). The Asp residues in motifs A and C coordinate two divalent metal ions that are essential for catalysis, typically Mg^2+^ or Mn^2+^. Motif F contains the positively charged residues Arg and Lys that mediate interactions with the triphosphate moieties of incoming nucleoside triphosphates (NTPs) (Butcher et al., 2001; Ng et al., 2008; Gong and Peersen, 2010; Lang et al., 2013). Motif G is located in the template cleft and is involved in protein primer orientation during the initiation of RNA replication (Gorbalenya et al., 2002; Ng et al., 2002).

**Table 2:** Conserved motifs and their functions.

Motif^∗^	Residue numbers^∗∗^	Function	References
G	123–134	Correct orientation of a template and a primer	Gorbalenya et al., 2002; Ng et al., 2002
F	173–191	Coordination of the triphosphate moiety of NTPs	Butcher et al., 2001; Ng et al., 2008; Gong and Peersen, 2010; Lang et al., 2013
A	250–259	M^2+∗∗∗^ coordination, NTP binding, catalysis	Ng et al., 2008; Choi, 2012
B	308–318	Template and NTPs positioning, selection of NTPs over dNTPs	Gohara et al., 2000; Ferrer-Orta et al., 2007; Gong and Peersen, 2010
C	353–355	M^2+^ coordination, NTP binding, catalysis	Kamer and Argos, 1984
D	373–376	NTPs binding, active site closure, export of PP_i_ from the active site, fidelity determination	Castro et al., 2007, 2009; Yang et al., 2012
E	400–404	Formation of NTPs entry tunnel, template and nascent strand binding	Poch et al., 1989; Jacobo-Molina et al., 1993; Han et al., 2017


*^∗^Motifs are listed according to their position in the protein, starting with the motif closest to the amino-terminus (N-terminus). ^∗∗^Amino acid positions refer to the RHDV RdRp (UniProt ID: P27411). ^∗∗∗^M, Metal.*

The thumb domain of calicivirus and picornavirus RdRps is small compared with that of other RdRps and DNA-dependent DNA polymerases. The domain consists of only four helices and forms a relatively large, 15 Å-wide central cleft (also named a channel) that leads to the active site (Ferrer-Orta et al., 2004, 2006). This cleft accommodates both the template and a VPg-linked primer (Choi, 2012).

The main function of RdRps is to copy RNA. This process is based on transferring the α-phosphate moiety of a complementary nucleotide to the 3′-OH end of the nascent strand. This reaction depends on two divalent metal ions (Mn^2+^ or Mg^2+^) in the active site. The metal ions are coordinated by the Asp residues of motifs A and C. One of the ions interacts with the 3′-OH group of the primer, which reduces the affinity of this group for the hydrogen and enables a nucleophilic attack of the negatively charged 3′-O^-^ on the α-phosphate residue of the incoming complementary nucleotide (Steitz, 1998). The second metal ion is involved in positioning the incoming NTP and the release of a pyrophosphate (PP_i_). As a result of the nucleophilic attack, a new phosphoester bond between the 3′-OH terminal group of the protein-linked primer and the α-phosphate of nucleoside monophosphate (NMP) is created and PP_i_ is released (Joyce and Steitz, 1995; Steitz, 1998).

## Structural and Functional Characteristics of Norovirus and Lagovirus RdRps

### Noroviruses

**FIGURE 4:**
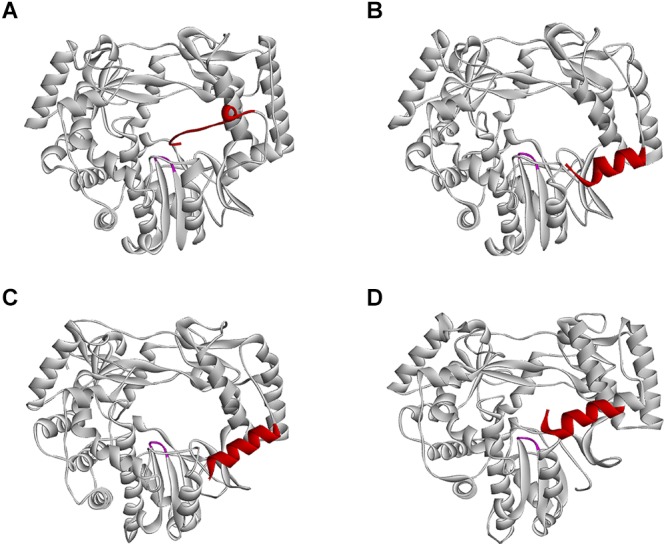
Position of the C-terminus in different calicivirus RdRps. **(A)** *Norwalk virus* (PDB ID: 1SH0); **(B)** MNV (PDB ID: 3NAH); **(C)** RHDV (PDB ID: 1KHW); **(D)** *Sapporo virus* (PDB ID: 1CKW) RdRps, presented as ribbon diagrams. C-terminal amino acids are colored red; the highly conserved motif C is colored magenta. Ribbon diagrams were generated using Discovery Studio (Dassault Systèmes BIOVIA, Discovery Studio Visualizer v17.2.0).

The overall structure of norovirus RdRps is similar to that of other caliciviruses, but some differences exist (Figure 4A–D). For example, the carboxyl terminus (C-terminus) of the protein is located within the active site cleft close to the two catalytic Asp residues (Ng et al., 2004; Figure 4A). Therefore, the C-terminus is suitably positioned to take part in the initiation of RNA replication. This configuration is similar to that in the RdRps of the *Hepatitis C virus* (HCV) and the φ6 bacteriophage, in which C-terminal amino acids help to stabilize primers in the active site (Butcher et al., 2001; Laurila et al., 2002; Ranjith-Kumar et al., 2002). This C-terminal addition to the active site would not allow the entry of RNA in the form of a duplex with a long primer, but it does not prevent an interaction of the template with a short dinucleotide primer (Ng et al., 2004). RNA binding to the active site of the norovirus RdRp also causes the rotation of the main helix of the thumb domain (residues 435–449) by 22°, thus forming a suitable groove for a protein-linked primer (Zamyatkin et al., 2008). Sapovirus RdRps share many features with those of noroviruses, e.g., the C-terminus of the sapovirus RdRp is located within the active site cleft (Fullerton et al., 2007; Figure 4D).

### Lagoviruses

Several lines of evidence suggest that functional lagovirus RdRps exist as a 3CD-like precursor protein and a mature protein. Both the *in vitro* translation of viral RNA with a subsequent precipitation of the products using region-specific antisera, as well as the *in vivo* analysis of proteins present in RHDV-infected primary hepatocytes revealed a 72 kDa protein corresponding to an uncleaved 15 kDa 3C-protease and 58 kDa polymerase (Martín Alonso et al., 1996; König et al., 1998). Subsequent *in vitro* studies with recombinant proteins suggest that this 3CD-like precursor possesses both protease and polymerase activities and is able to uridylate VPg (Machín et al., 2009).

**FIGURE 5:**
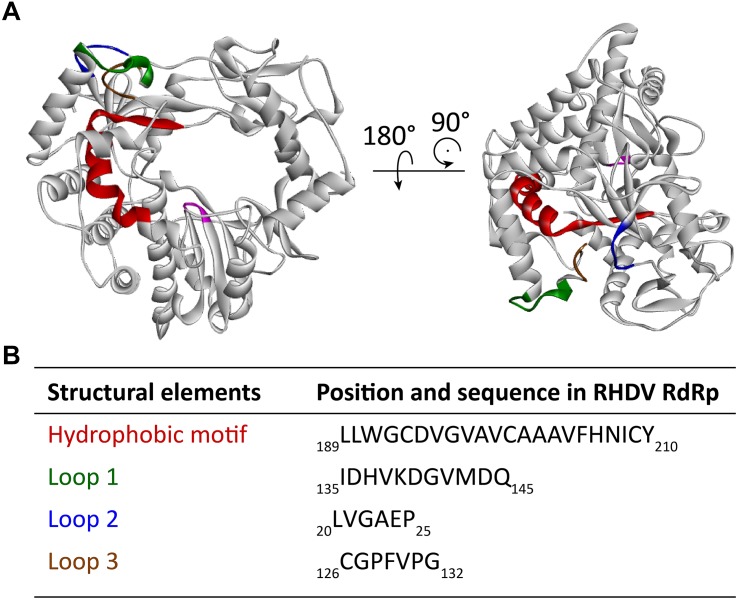
Localization of a partially buried hydrophobic membrane interaction motif in the RHDV RdRp. **(A)** Ribbon diagrams of the RHDV RdRp (PDB ID: 1KHW). The hydrophobic motif is colored red, loop 1 green, hydrophobic loop 2 blue, and hydrophobic loop 3 brown. The active site (motif C) is highlighted magenta to provide a reference point for the position of the hydrophobic motif in the RdRp. **(B)** Amino acid positions and sequences of the structural elements highlighted in the diagrams above. Ribbon diagrams were generated using Discovery Studio (Dassault Systèmes BIOVIA, Discovery Studio Visualizer v17.2.0).

Many RNA viruses, including caliciviruses, use cellular membranes to protect and act as a scaffold for their RNA replication machinery (Green et al., 2002). A number of viral proteins recruit intracellular membranes (e.g., p48 of Norwalk virus) but polymerases are usually not involved. One of the most remarkable findings with lagovirus RdRps is their apparent ability to interact with intracellular membranes and to change the architecture of the Golgi apparatus. The expression of recombinant RHDV and RCV RdRps induced a striking rearrangement of *cis*/medial and medial/*trans* Golgi membranes (Urakova et al., 2015, 2017a). However, all immunofluorescence studies on the intracellular localization of the recombinant lagovirus RdRps that have been conducted so far have failed to detect a colocalization of RdRps with Golgi (or other) intracellular membranes (Urakova et al., 2015, 2017a). Furthermore, the overexpression of recombinant proteins without viral replication may result in more RdRp proteins being available to change the localization of Golgi membranes (as compared to the situation in virus-infected cells). This may explain why barely detectable amounts of RdRps were observed to be sufficient to induce dramatic changes to the Golgi apparatus (Urakova et al., 2015, 2017b). The enzymatic activity of the RdRp is not required for the RdRp to disaggregate the Golgi apparatus, as active site (motif C) variants with Gly-Asp-Asp to Gly-Asn-Asp and Gly-Asp-Asp to Gly-Ala-Ala substitutions had the same effect on Golgi membranes as proteins with the wild type sequence (Urakova et al., 2017a). The observed Golgi membrane disruption is most likely a consequence of cellular membrane recruitment for the formation of a membranous vesicle network on which virus replication occurs, similar to the membrane recruitment in other caliciviruses and picornaviruses (Schlegel et al., 1996; Green et al., 2002). A hydrophobic motif (residues 189–210) that might be responsible for the interaction with Golgi membranes has been identified (Urakova et al., 2017b; Figure 5A). This motif is located next to the F motif within the F homomorph (this newly identified hydrophobic motif should not be confused with the “classic” conserved motifs A to G). Truncated RHDV RdRp variants without the hydrophobic motif showed a diffuse cytoplasmic localization when expressed in transiently transfected cells. None of these variants accumulated in the distinct cytoplasmic foci that are typical for the intracellular localization of the wild type RdRp (Urakova et al., 2017b). Furthermore, the hydrophobic motif is able to change the localization pattern of other proteins, as it has been demonstrated using the green fluorescent protein (Urakova et al., 2017b). A similar hydrophobic motif was observed in the RdRp of RCV, also in the F homomorph and in the same position as in the RHDV RdRp, but the motif does not exist, or is less obvious in more distantly related caliciviruses (Urakova et al., 2017b). The importance of the hydrophobic amino acids within the motif was demonstrated using variants in which individual Val residues were changed to Ser residues. A variant with two Val to Ser substitutions in the C-terminal part of the motif exhibited a diminished ability to rearrange Golgi membranes, and a variant with four such mutations completely lost this feature (Urakova et al., 2017b).

Research into the newly identified hydrophobic motif revealed an unexpected structural flexibility of calicivirus RdRps, as the exposure of the partially buried hydrophobic motif requires a series of conformational changes. Molecular dynamics simulations suggest that four regions surrounding the motif possess a high degree of mobility (loops 1–3 in Figure 5B). Two of these loops (loops 1 and 2) flank the “point of access” to the motif, and the third loop covers the motif, much like a “trap door.” The following sequence of movements is thought to bring the RdRp close to an intracellular membrane and allow exposure of the hydrophobic motif (Urakova et al., 2017b): firstly, three collinear, positively charged Lys residues at the edge of a solvent-exposed helix next to the loop 1 interact with the negatively charged surface of the membrane. Next, hydrophobic interactions, including those between the partially hydrophobic loop 2 and the membrane, draw the protein further toward the membrane to a point, at which hydrophobic loop 3 makes contact with the membrane, moves out of the way, and allows the hydrophobic motif to become exposed and to insert itself into the membrane.

## Genomic and Subgenomic RNA Replication

Detailed studies on calicivirus replication and pathogenicity often lag behind those in other RNA virus families. For decades, studies on human norovirus and other enteric caliciviruses have been hampered by the lack of a robust cell culture system. Of note, it has been reported that replication competent RHDV RNAs can be generated from plasmids using a T7 promotor and a hepatitis D virus ribozyme (Zhu et al., 2017), but these findings have not yet been independently reproduced. Very recently, however, ground-breaking progress was made in enteroid cultivation methods that show great potential for providing new cell culture systems for noroviruses and lagoviruses (Jones et al., 2015; Ettayebi et al., 2016). The new methods complement and supplant previously developed cell culture models for MNV that relied on bone marrow-derived murine macrophages and dendritic cells. These MNV cell cultures were used as surrogate models to study human noroviruses (Wobus et al., 2004, 2006). However, there is still no general agreement on certain steps of the calicivirus replication process, such as the mechanism of the replication initiation.

### Terminal Transferase Activity of RdRps

Terminal transferase activity is the ability to add nucleotides to the 3′ end in a template independent manner. Similar to poliovirus (Arnold et al., 1999) and HCV RdRps (Ranjith-Kumar et al., 2001), human norovirus RdRps possess terminal transferase activity (Rohayem et al., 2006a). The activity is thought to serve as a repair system for 3′ ends that were damaged by cellular exonucleases and, in some cases, it facilitates the initiation of RNA synthesis through the addition of non-templated nucleotides (Wu and Kaper, 1994). For example, the terminal adenylyl transferase activity of the poliovirus 3D polymerase restores the infectivity of poliovirus RNA genomes that lack a poly(A) tail (Neufeld et al., 1994). The terminal transferase activity of calicivirus RdRps generates not only a protective poly(A) tail but may also generate a poly(C) tail that has been suspected to play a critical role in the initiation of genomic and subgenomic RNA synthesis.

### Initiation of RNA Synthesis

**FIGURE 6:**
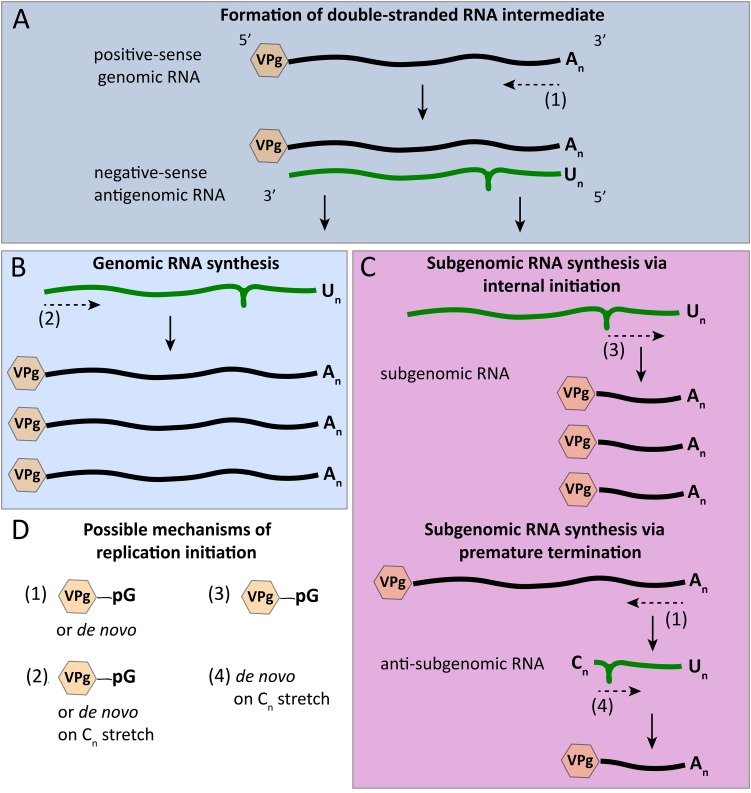
Initiation modes for RNA synthesis during calicivirus replication. **(A)** The synthesis of antigenomic RNA results in the formation of a double-stranded RNA intermediate; antigenomic RNA synthesis is initiated in a VPg-dependent manner or *de novo*. **(B)** The synthesis of new genomic RNA was described to start either *de novo* or from a poly(C) stretch of nucleotides that were added by the RdRp’s terminal transferase activity. **(C)** The synthesis of subgenomic RNA may be initialized internally using a stem loop in the negative-sense antigenomic RNA and VPg priming; according to an alternative mechanism, a premature termination of antigenomic RNA synthesis results in anti-subgenomic RNA that is then used as a template for subgenomic RNA synthesis, a process that is suggested to involve a poly(C) stretch similar to the proposed initiation of genomic RNA synthesis. **(D)** Overview of the various mechanisms that were postulated for the initiation of calicivirus RNA synthesis. Green and black lines symbolize negative- and positive-sense RNAs, respectively; the loop in negative-sense RNAs indicates the position of a stem loop that may act as a subgenomic promoter region; dashed arrows indicate the initiation point and direction of RNA synthesis; hexagons represent VPg proteins that are covalently bound to the 5′ end of all positive-sense RNAs; pG indicates guanylation; A_n_, U_n_, and C_n_ represent poly(A), poly(U), and poly(C) sequences, respectively.

In calicivirus replication, the initiation of RNA synthesis occurs differently for genomic and antigenomic RNAs (Figure 6A,B,D). The former may occur in a primer independent manner (Rohayem et al., 2006b), and the latter is generally thought to depend on a nucleotidylated VPg primer. However, according to an article by Subba-Reddy et al. (2012), the synthesis of negative-sense RNAs can be initiated *de novo.* This article was later retracted (Subba-Reddy et al., 2017), although the authors maintain that the overall conclusion in the original article is still valid (“[…] that the norovirus major capsid protein could modulate norovirus RdRp activity has been confirmed in both the Kao and the Goodfellow labs”). Clearly, more research is warranted to better understand the initiation of norovirus RNA synthesis. Interestingly, the VPg of human noroviruses consists of 133 amino acids, which is substantially larger than the 22 amino acids of the homologous protein in picornaviruses. Nevertheless, the larger size does not prevent the norovirus VPg from serving as a protein primer. As shown by Rohayem et al. (2006b) in a series of *in vitro* experiments, the norovirus RdRp is able to initiate synthesis on subgenomic polyadenylated RNA that carries a VPg protein in the absence of a short poly(U) RNA primer but in the presence of VPg, confirming the notion of a protein-primed initiation. For anti-subgenomic RNA replication to occur, a protein primer is probably not required, as an RNA product is yielded in the absence of exogenous primers (Rohayem et al., 2006b). Interestingly, a poly(C) stretch was detected at the 3′ terminus of newly produced anti-subgenomic norovirus RNA, which may have been the result of a terminal transferase activity of the norovirus RdRp (Figure 6C). Based on these findings, it was proposed that the initiation of subgenomic RNA synthesis occurs *de novo* on a short 3′-terminal poly(C) stretch of anti-subgenomic RNA, a scenario that is in line with the general observation that the *de novo* initiation on RNA genomes usually starts on pyrimidines (Kao et al., 2001; Rohayem et al., 2006b). However, other evidence suggests that the synthesis of positive-sense genomic and subgenomic RNAs also depends on a VPg primer, since infectious (positive-sense) RNAs isolated from MNV-infected cells are linked to VPg (Chaudhry et al., 2006). Similar *in vitro* studies were performed using the 3′-terminal region of genomic norovirus RNA. In these studies, the norovirus RdRp was shown to initiate the synthesis of antigenomic RNA primer-independently (Fukushi et al., 2004). Moreover, a 3′-terminal region without the poly(A) sequence was replicated as effectively as RNA with the original sequence, indicating that a poly(A) sequence is not necessary for the initiation of negative strand RNA synthesis (Fukushi et al., 2004). With sapoviruses, initiation of RNA synthesis was also observed to be different for subgenomic polyadenylated RNA and anti-subgenomic RNA. In the case of subgenomic RNA synthesis, replication initiation is primer independent, whereas the synthesis of anti-subgenomic RNA is strictly primer dependent and occurs only when an oligo(U) primer is added to the reaction (Fullerton et al., 2007). Terminal transferase activity was also shown to be a feature of sapovirus RdRps (Fullerton et al., 2007).

### Binding of Promoter Regions and Other RdRp-RNA Interactions

The initiation of RHDV subgenomic RNA replication was studied in great detail and these observations may guide a better understanding of calicivirus promoters. There are two possible mechanisms for the synthesis of subgenomic RNA (Figure 6C). It can either be through an internal initiation on a negative strand of genomic RNA, or through a premature termination of genomic negative strand RNA synthesis. The latter would result in negative-sense subgenomic RNA that can be used as a template for positive-sense subgenomic RNA production (Sit et al., 1998; Miller and Koev, 2000). Subgenomic RNA replication in RHDV was shown to be initialized internally on negative strand genomic RNA, and a suitable promoter region was discovered upstream of the subgenomic RNA synthesis start site (Morales et al., 2004). The localization and extent of this subgenomic RNA promoter region was analyzed by constructing deletion mutants with truncated 3′-terminal sequences on the negative strand genomic RNA. At least 50 nucleotide residues preceding the start of the subgenomic RNA were required for subgenomic RNA production (Morales et al., 2004).

Subsequent studies revealed a stable and evolutionarily conserved stem-loop in the negative strand of genomic RNA of all caliciviruses that is located six nucleotides upstream of the start of the subgenomic RNA in the RdRp coding region (Simmonds et al., 2008). The role of this stem loop in subgenomic RNA synthesis was studied by the introduction of nucleotide substitutions in the stem-loop sequence of an MNV replicon that contained the *Renilla* luciferase gene fused to the foot-and-mouth disease virus (FMDV) 2A protease coding sequence ahead of the VP2-coding region. These reporter replicon variants were used to quantify subgenomic RNA synthesis. Replicons with mutations in the stem-loop produced less luciferase compared with wild type MNV replicons, but similar amounts to a replication-defective replicon. The amount of subgenomic RNA was determined using a primer extension assay, in which a radiolabeled primer complementary to the 5′ region of subgenomic RNA was used to generate a product corresponding to the start of the subgenomic RNA. Subgenomic RNA was detected in cells transfected with the wild type MNV genome but was absent in those transfected with a replicon bearing mutations in the stem-loop region. These results confirm the hypothesis that the stem-loop in the RdRp coding region is essential for the initiation of subgenomic RNA synthesis (Yunus et al., 2015).

In the search for the protein region that is involved in RNA recognition and binding, several amino acid residues of the MNV RdRp that potentially interact with genomic RNA were identified: Lys169, Lys183 and 184, Arg185, Lys210, Arg395, and 396, and Lys422. These positively charged amino acid residues are located adjacent to the active site and well conserved across the *Caliciviridae* family. Using site-directed mutagenesis, seven MNV variants were created, in which positively charged amino acids were substituted with a non-polar Ala (Han et al., 2017). The effect of these substitutions on protein-RNA interactions was examined using electrophoretic mobility shift assays, and the impact of these substitutions on RNA replication was studied in cell culture. The results demonstrate that RdRp variants with Ala substitutions interact with the RNA less efficiently and are either non-viable or replicate with reduced efficiency (Han et al., 2017).

Finally, the FCV polymerase-protease precursor protein was found to interact with the ORF2 region of the viral genome. ORF2 encodes the major structural protein VP1 and the LC protein. This interaction is suspected to be required for the encapsidation of the viral RNA, although this is yet to be proven (Kaiser, 2006).

### RdRp-Mediated VPg Nucleotidylation

VPg nucleotidylation is catalyzed much more efficiently by the human norovirus protease-polymerase precursor protein than by the mature enzyme (Medvedev et al., 2017). While nucleotidylation by the precursor protein occurs without a poly(A) template, the mature RdRp requires such a template (Rohayem et al., 2006b; Belliot et al., 2008). Unlike the poliovirus protease-polymerase precursor 3CD that shows only protease activity, the homolog of human noroviruses possesses both protease and polymerase activities, is able to initiate RNA synthesis, and can elongate the nascent RNA (Belliot et al., 2005). The FCV RdRp precursor protein was also suspected to be an active polymerase, because infected cells contain more of the uncleaved precursor than the mature enzyme (Sosnovtseva et al., 1999). Subsequent experiments confirmed that the precursor indeed has polymerase activity. The deletion of 164 amino acids from the amino terminus of the precursor only caused a threefold decrease in polymerase activity, but the deletion of the next amino acid resulted in a 90-fold reduction (Wei et al., 2001). This study defines the boundary of the active form of the FCV RdRp that is located either at Val135 or further toward the amino terminus (Wei et al., 2001).

During FCV replication, a direct interaction of the VPg with the polymerase-protease precursor protein was detected in an ELISA-based assay, in which purified VPg was adsorbed to the wells and the recombinant precursor protein was passed over (Leonard et al., 2000; Kaiser, 2006). The results support the idea of a protein-primed initiation of replication, a concept that was further validated by research into RHDV replication. The RdRp of RHDV also transfers nucleotides to VPg (Machín et al., 2001). Moreover, the RHDV RdRp precursor (p72) catalyzed VPg uridylation more actively than the mature enzyme, although the mature form showed a higher *in vitro* polymerization activity when a heteropolymeric RNA was used as a template (Machín et al., 2009). Mutational analysis of the FCV VPg further confirmed the hypothesis of a protein-primed initiation of replication: the substitution of Tyr24 to Ala (as well as to Thr, Phe, and Ser) was lethal for the virus (Mitra et al., 2004). A Tyr in this position is believed to be essential for the VPg uridylation in FCV, similar to Tyr21 in the RHDV VPg. The critical amino acid in the RHDV VPg was detected by the deletion of the first 21 N-terminal residues, which completely stopped uridylation. When Tyr21 was substituted either by Phe, Ser, or Thr, the resulting variants were no longer uridylated, due to steric hindrances (in the case of Ser and Thr substitutions), or the lack of a hydroxyl group (in the case of Phe) that is needed as a nucleophile in the uridylation reaction (Machín et al., 2001).

The substrate specificity of RdRps varies. For example, human norovirus RdRps nucleotidylate only human norovirus VPgs, whereas the RdRp of MNV efficiently nucleotidylates the VPgs of both human and murine noroviruses (Min et al., 2012). For the MNV RdRp, this reaction can be enhanced by *in vitro*-transcribed positive and negative strand subgenomic RNAs. Of all RNAs tested, it was the ORF3 region of the subgenomic negative strand RNA that stimulated nucleotidylation most effectively, indicating that the ORF3 region contains a *cis-*acting element that stimulates the reaction (Han et al., 2010).

## Interactions of RdRps and Other Proteins

### Viral Interaction Partners

Many calicivirus protein-protein interactions have been investigated using MNV, because this virus can be propagated in cell culture (Wobus et al., 2004). VPg clearly needs to interact with the calicivirus RdRp. However, this interaction also occurs independent of VPg-priming, because VPg variants that lack the Tyr residue needed for the nucleotidylation process still enhanced the replication process *in vitro* (Lee et al., 2018).

Further protein-protein interactions were detected using a cell-based assay in which the human norovirus GII.4 RdRp was assessed for its ability to synthesize RNA (Subba-Reddy et al., 2011). The assay uses the ability of various cellular pattern recognition receptors, such as the retinoic acid-inducible gene I (RIG-I) to detect viral RNA to activate and the expression of interferon (IFN)-regulated genes (Patel et al., 2003; Honda et al., 2005). Measuring the luciferase production that is driven by an IFN-β promoter can thus be used to quantify viral RdRp activity, as an increasing virus RNA concentration correlates with an increasing expression of IFN-regulated genes (Subba-Reddy et al., 2011). Using this rather indirect reporter assay, Subba-Reddy and coworkers investigated which viral proteins had stimulatory or inhibitory effects on RNA replication. The researchers reported a stimulatory effect for the human norovirus non-structural protein p48 and the structural protein VP1, and an inhibitory effect for VP2. But when these GII.4 proteins were co-expressed with the RdRps of other viruses, they did not significantly increase virus RNA replication (again quantified via the expression of the IFN-β-dependent reporter), suggesting that these calicivirus protein-protein interactions occur in a species-specific manner (Subba-Reddy et al., 2012, 2017). Further experiments identified the part of the VP1 protein that is responsible for the interaction with RdRp. VP1 consists of two domains, a shell domain and a protruding domain. The co-expression of the RdRp with a series of truncated VP1 proteins revealed that the shell domain was sufficient to modulate the enzyme activity. The findings were confirmed using MNV replicons: the transfection of cells with a replicon defective for VP1 expression showed impaired replication, but when VP1 expression was restored by *in trans*-complementation, virus replication was rescued. Presumably, this positive feedback (the more positive-sense RNA is synthesized, the more VP1 is translated) slows at the point when VP1 starts to multimerize and assemble into new capsids, which prevents its interaction with RdRp and stops the stimulation of RNA synthesis (Subba-Reddy et al., 2012, 2017).

### Cellular Interaction Partners

Only caliciviruses that grow in cell culture, such as FCV and MNV (Wobus et al., 2006; Vashist et al., 2009), allow investigations of RdRp interactions with cellular proteins during genuine virus replication. A redistribution of nucleolin from the nucleoli to the nucleoplasm as well as the perinuclear area was observed in FCV-infected cells. Subsequent studies showed that the FCV RdRp directly interacts and colocalizes with nucleolin, and that this interaction is necessary for efficient virus replication. Given that nucleolin interacts with both RdRp and the 3′ UTR of viral RNAs, it has been suggested that the interaction promotes the formation of replication and/or translation complexes (Cancio-Lonches et al., 2011).

The FCV protease-polymerase precursor inhibits host gene transcription mediated by the cellular RNA polymerase II. The effect was observed using reporter genes under the control of either an endogenous promoter (in this case, the feline IFN-β promoter) or exogenous promoters (simian virus 40, cytomegalovirus, or bacteriophage T7 promoters). Moreover, a domain was identified in the N-terminal region of the protease-polymerase precursor that is responsible for the observed inhibition of the RNA polymerase II (Wu et al., 2016). Similarly, the poliovirus 3C protease shuts off cellular transcription through the cleavage of the TATA-binding protein, which prioritizes the synthesis of viral proteins (Kundu et al., 2005).

Another cellular protein that affects FCV, MNV, and porcine enteric calicivirus (PEC) replication is the lysosomal endopeptidase cathepsin L, a protease that is involved in apoptosis and is primarily located in endosomes. Cathepsin L cleaves the structural protein VP1 of FCV and MNV, and VP2 of PEC. Its inhibition was shown to negatively affect the replication of FCV, MNV, and PEC in cell culture. The effect of cathepsin L inhibition is similar to the inhibition of endosomal acidification (a necessary step during viral entry) and prevents MNV and PEC from endosomal escape. These and possibly other caliciviruses enter host cells via clathrin-mediated endocytosis, thus, it should not come as a surprise that any interference with the endosomal escape of incoming virus particles blocks the initiation of virus replication (Shivanna et al., 2014a,b).

### Co- and Post-translational Modifications of Calicivirus RdRps

Co- and post-translational modifications refer to a process in which a protein undergoes enzymatically driven covalent modifications during or following translation. At least some calicivirus RdRps are modified in that manner, e.g., the signaling kinase Akt phosphorylates the norovirus RdRps at residue Thr33 (located at the interface between finger and thumb domains) (Eden et al., 2011). Akt is a serine/threonine protein kinase involved in multiple cellular pathways; it promotes survival through the inhibition of apoptosis and the regulation of the cell cycle (Datta et al., 1999). The consequences of RdRp phosphorylation were studied by comparing the kinetic properties of the wild type enzyme to those of a Thr33 to Glu variant that mimics phosphorylation (Eden et al., 2011). In a *de novo* GTP incorporation assay that can be used to analyze enzyme kinetics (Bull et al., 2010b), the Thr33 to Glu variant showed a lower maximum enzyme velocity (100 vs. 125 fmol × min^-1^) and had a lower affinity for the GTP substrate than the wild type, suggesting that phosphorylating Thr33 modulates the activity of the enzyme (Eden et al., 2011).

### Oligomerization of RdRps

Norovirus RdRps were shown to form homodimers (Högbom et al., 2009), a phenomenon that had already been described for picornavirus RdRps (Lyle et al., 2002). When different amounts of purified recombinant norovirus RdRp protein were subjected to PAGE in native (non-denaturing) conditions, dimer formation was observed at high protein concentration and subsequently confirmed by a denaturation of the isolated proteins, SDS-PAGE, and Western blotting. The formation of dimers seems to be of biological importance, as norovirus RdRps demonstrate cooperative enzymatic activity. Increasing amounts of RNA can be synthesized *in vitro* with increasing concentrations of RdRp, until a plateau phase is reached. Högbom and coworkers interpret the data as a shift from active monomeric RdRps to more active dimers. They analyzed the cooperativity between RdRp monomers by calculating the Hill coefficient that, in this case, was determined to have a value greater than one, which is indicative of a positive cooperativity (Högbom et al., 2009).

In MNV, the interaction of RdRp with VPg stimulates RdRp multimerization and formation of large fibril-like structures, a reaction that can be observed by transmission electron microscopy (Lee et al., 2018). Analysis of the crystal structure of the MNV RdRp together with a truncated VPg (consisting of the first 73 amino acids) suggested that two amino acid residues of the RdRp, Asp331, and Leu354, might be involved in the interaction between RdRp and VPg. When Asp331 was changed to Ala and Leu354 to Asp, the resulting RdRp variants were still able to form hexamers in the absence of VPg, but did no longer form higher order protein structures in the presence of VPg. Moreover, the binding affinity of these variants to full length VPg decreased significantly, confirming that Asp331 and Leu354 are critical for the interaction of RdRp with VPg. It has been speculated that the formation of RdRp multimers and tubular fibrils may lead to a better coordination of replication components within larger clusters and thus enhance replication efficiency (Lee et al., 2018).

## Enzymatic Properties of Calicivirus RdRps

### Polymerase Fidelity, Replication Speed, and Evolutionary Rates

Calicivirus RdRps, as well as the RdRps of other RNA viruses are known to be error-prone enzymes, because they lack the proofreading activities of many DNA polymerases. Approximately one error occurs per replication cycle for RNA viruses compared with one error per 300 cycles for DNA viruses (Drake, 1991, 1993). Comparing studies with different error reporting units is somewhat challenging, but certain trends emerge. The average error rate for HCV (family *Flaviviridae*) is 3.8 × 10^-5^, measured as substitutions per nucleotide per cycle of infection (s/n/c) (Sanjuán and Domingo-Calap, 2016; Selisko et al., 2018), and the error frequency of the poliovirus RdRp ranges from 7 × 10^-4^ to 5.4 × 10^-3^, as determined by the ratio of non-complementary nucleotides incorporation to the total number of nucleotides (Ward et al., 1988). Similar RdRp error rates were determined for several viruses of the family *Caliciviridae*, e.g., 6.8 × 10^-4^ for MNV, 1.6 × 10^-4^ for sapovirus GI, and 9.0 × 10^-4^ nucleotide substitutions/site for norovirus GII.4 (Bull et al., 2010b).

RNA-dependent RNA polymerase properties, such as fidelity and replication rate, are important factors that shape virus evolution. For example, RdRps from norovirus GII.4 strains had higher mutation rates (determined using *in vitro* fidelity assays) compared with those of the closely related but less frequently detected GII.b and GII.7 strains (5.5–9.1 × 10^-4^ substitutions per site for GII.4 RdRps vs. 1.5 × 10^-4^ and 2.2 × 10^-5^ substitutions per site for GII.b and GII.7, respectively). Interestingly, the GII.4 lineage showed an approximately 1.7-fold higher rate of evolution of capsid sequences and a higher frequency of non-synonymous changes compared with non-pandemic norovirus strains (Bull et al., 2010a). Furthermore, Mahar et al. (2013) reported that the acquisition (by recombination) of new GII.3 RdRp variants with higher mutation rates may increase genetic diversity and improve the overall fitness of viral populations under selective pressures. Taken together, a low fidelity rate seems to correlate with a higher evolutionary rate.

The replication rate of a virus is another determinant of viral fitness, since viruses with an increased replication rate can produce more copies of their genome, which would result in more variants even if the RdRp error rate remains the same. For example, the RdRps from the 2006 GII.4 pandemic strains had a higher nucleotide incorporation rate (i.e., they replicated faster) than the recombinant GII.4 RdRps from earlier outbreaks and the US95/96-like pandemic GII.4 strain although the error rates were very similar. The observed increase in the incorporation rate has been associated with the appearance of a mutation outside of the active site, i.e., a Lys291 to Thr substitution in the RdRp finger domain (Bull et al., 2010a). Thus, high mutation and/or replication rate within the GII.4 lineage seem to correlate with the evolution of pandemic strains. However, high replication rates do not always correlate with a high overall fitness of a virus, which suggests that speed needs to be balanced with suitable mutation rates. For example, the GII.7 norovirus lineage, despite having a high replication rate, has a low mutation rate and limited geographic spread (Bull et al., 2010a). It is possible that the speed at which this particular virus replicated was not fast enough to balance its limited ability to produce new variants through the incorporation of mutations.

The contribution of the RdRp to the evolutionary rate of caliciviruses became even more clear with the recent success of recombinant GII.2 and GII.4 viruses that acquired a new polymerase variant. For example, the reemerged recombinant norovirus GII.P16-GII.2 that differs from previous GII.P16-GII.2 strains by 5 amino acids in the RdRp (Ruis et al., 2017), results in high virus loads in feces, possesses a relatively high evolutionary rate (5.5 × 10^-3^ substitutions/site/year), and has rapidly spread across the world (Ao et al., 2018; Cheung et al., 2019). It has been suggested that the amino acid changes in the new RdRp affect the kinetic properties and the fidelity of the enzyme, but the exact mechanistic details remain unknown.

Genetic recombination events have also been observed between different lagoviruses. RHDV2, originally a virus with moderate virulence and limited geographical range (Le Gall-Reculé et al., 2013), appears to have evolved into a more virulent virus (Capucci et al., 2017), a change that is believed to be at least partially a consequence of recombination with other lagoviruses (Lopes et al., 2015). Some of these recombinant viruses were found to possess the non-structural proteins of benign rabbit calicivirus Australia-1 (RCV-A1)-like viruses (Lopes et al., 2015; Hall et al., 2018). RHDV and RCV-A1 have evolutionary rates of 2.8 × 10^-3^ and 5.0 × 10^-3^ substitutions/site/year, respectively (Eden et al., 2015; Mahar et al., 2016). The higher evolutionary rate of RCV-A1 correlates with a higher speed of its RdRp, as determined by *in vitro* assays (Urakova et al., 2016). It is tempting to speculate that RHDV2 could have acquired a relatively fast polymerase, which may explain its increased virulence and apparent evolutionary success. Within 18 months of its arrival, RHDV2 largely replaced endemic RHDV strains in Australia (Mahar et al., 2017).

The generation of a genetically highly diverse pool of genomes provides an evolutionary advantage, because a diverse virus population can more readily adapt to selective pressures (Domingo, 2002; Lauring and Andino, 2010). If the diversity is the result of a higher error rate, this can also increase the likelihood of acquiring detrimental mutations and it has therefore been suggested that most RNA viruses replicate at the edge of an error threshold that is determined by a complex interplay of several parameters such as genome size, error rates, and replication speed (Duffy et al., 2008). As such, it should not come as a surprise that both increases and decreases in RdRp fidelity can affect viral fitness (Pfeiffer and Kirkegaard, 2005; Xie et al., 2014; Arias et al., 2016; Agol and Gmyl, 2018).

### Effects of Temperature, pH, and Salt Conditions on RdRp Performance

The conditions for an optimal performance of calicivirus RdRps were determined for viruses from the genera *Norovirus*, *Sapovirus*, and *Lagovirus* (Table 3). The activity of viral RdRps is temperature dependent, although the optimal temperature is not necessarily that of the host’s body. In early studies, the highest sapovirus RdRp activity was detected at 37°C (Fullerton et al., 2007). However, more recent studies indicate that many calicivirus RdRps work in an environment that does not allow for maximal performance. For example, a human norovirus RdRp demonstrated a higher activity at 30 than at 37°C according to *in vitro* assays (Rohayem et al., 2006a). Moreover, when a broader temperature range was studied (i.e., 5, 25, 37, 55, 65, and 75°C) with human norovirus and sapovirus RdRps, the activity was highest at 25°C, and only about 50% of the optimal enzymatic activity was exhibited at 37°C (Bull et al., 2010b). Furthermore, the norovirus and sapovirus RdRps displayed only approximately 20% of their optimal activity at 5°C and only about 1% at 55°C. No activity was detected at 65 or 75°C for any of the RdRps except sapovirus RdRp, which still exhibited 13% of the optimal activity at 65°C (Bull et al., 2010b). Interestingly, the optimal temperature for some if not all lagoviruses is higher than that of human noroviruses and sapoviruses. Using recombinant proteins, it was found that the RdRps of the non-pathogenic RCV and the highly pathogenic RHDV performed best between 40 and 45°C (Urakova et al., 2016), a feature that can be explained as an adaptation of rabbit caliciviruses to their hosts, as the body temperature of healthy rabbits ranges from 38.3 to 39.4°C. Moreover, the fever associated with rabbit haemorrhagic disease often raises the body temperature to 42°C (Strive et al., 2010), but this temperature is not high enough to slow down the activity of the RHDV RdRp (Urakova et al., 2016). The reason why caliciviruses other than lagoviruses seem to possess a temperature optimum that is different from the core body temperature of the host is presently unknown and further research is required to answer this question.

**Table 3:** Enzymatic properties of calicivirus RdRps.

Genera	pH optimum	Me^2+^ preference (test conditions)	Temperature optimum (°C)	References
*Norovirus*	7.0–8.0	Mn^2+^ (2.5 mM MnCl_2_)	25	Bull et al., 2010b
		Mg^2+^ (0.5–1.5 mM MgCl_2_)	30	Rohayem et al., 2006a
			35–39	Urakova et al., 2016
*Lagovirus*	8.5	Mn^2+^ (2.5 mM MnCl_2_)	40–45	Vazquez et al., 1998
		Mg^2+^ [3 mM Mg(CH_3_COO)_2_]		Urakova et al., 2016
*Sapovirus*	8.0	Mn^2+^ (0.5–5 mM MnCl_2_)	25	Bull et al., 2010b
			37	Fullerton et al., 2007


The optimal pH for rabbit calicivirus RdRps was found to be 8.5, which is higher than that of norovirus RdRps (7.0–8.0) (Bull et al., 2010b; Urakova et al., 2016). For optimal catalytic function, the norovirus and lagovirus RdRps can utilize either Mn^2+^ or Mg^2+^, but not Fe^2+^ (Vazquez et al., 1998; Rohayem et al., 2006a; Urakova et al., 2016). Sapovirus RdRp demonstrated a higher activity with Mn^2+^, but it was also active when Mg^2+^ was added as a cofactor to the reaction, indicating some flexibility in the use of cofactors (Fullerton et al., 2007).

## A Putative Undescribed Conserved Motif in Calicivirus RdRps

**FIGURE 7:**
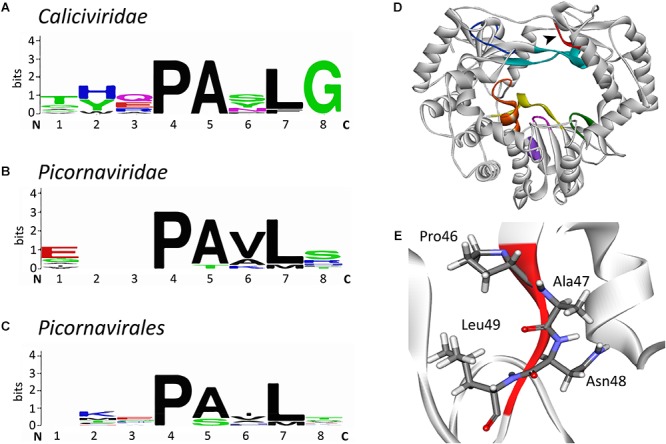
Sequence alignment logos of a putative new conserved motif (“motif I”) and the localization of the motif in the RHDV RdRp. **(A)** Sequence logo alignment for the putative motif of the following viruses in the family *Caliciviridae*: *European brown hare syndrome virus* and *Rabbit haemorrhagic disease virus* (both genus *Lagovirus*)*; Norwalk virus, Lordsdale virus*, *Murine norovirus* (genus *Norovirus*); *Sapporo virus* (genus *Sapovirus*); *Feline calicivirus*, *Vesicular exanthema of swine virus*, and *San Miguel sea lion virus* (genus *Vesivirus*); *Newbury 1 virus* (genus *Nebovirus*). **(B)** Sequence logo alignment for the putative motif of the following viruses in the family *Picornaviridae*: *Poliovirus*, *Bovine enterovirus, Coxsackievirus B3, Human rhinovirus A*, and *Echovirus* (genus *Enterovirus*); *Foot and mouth disease virus* (genus *Aphtovirus*); *Hepatitis A virus* (genus *Hepatovirus*); *Human parechovirus* (genus *Parechovirus*); *Theiler’s murine encephalomyelitis virus* and *Encephalomyocarditis virus* (genus *Cardiovirus*); *Avian encephalomyelitis virus* (genus *Tremovirus*). **(C)** Sequence logo alignment for the putative motif of the following viruses in the order *Picornavirales: Poliovirus, Foot and mouth disease virus*, *Hepatitis A virus*, and *Human parechovirus* (family *Picornaviridae*); *Cricket paralysis virus* and *Drosophila C virus* (family *Dicistroviridae*); *Parsnip yellow fleck virus, Broad bean wilt virus, Cowpea mosaic virus, and Beet ringspot virus* (family *Secoviridae*). N and C indicate N- and C-terminal directions, respectively. Sequence conservation is measured in bits and is indicated by the height of each letter’s stack. Amino acids are colored according to their chemical properties: polar amino acids (Gly, Ser, Thr, Tyr, Cys, Gln, Asn), green; basic (Lys, Arg, His), blue; acidic (Asp, Glu), red; and hydrophobic (Ala, Val, Leu, Ile, Pro, Trp, Phe, Met), black (Crooks et al., 2004). **(D)** Ribbon diagram of *Rabbit haemorrhagic disease virus* RdRp (PDB ID: 1KHW). The black arrow head points at the new motif I that is colored red, other conserved motifs are colored as in Figure 3D. **(E)** Structure of the motif I. Sequence alignments were performed with the multiple sequence alignment tool MUSCLE (Edgar, 2004); sequence logo pictures were created with Weblogo (Crooks et al., 2004). The ribbon diagram was generated using Discovery Studio (Dassault Systèmes BIOVIA, Discovery Studio Visualizer v17.2.0).

Our own sequence comparison of calicivirus RdRps revealed a conserved motif that had not previously been described. This short motif in the RHDV RdRp is located in the thumb domain and consists of four amino acids: _46_Pro-Ala-Asn-Leu_49_ (Figure 7D,E). The flanking amino acids Pro and Leu are significantly conserved, whereas the internal Ala and Asn are not (Figure 7A–C). This motif is present in all calicivirus and picornavirus RdRps, but does not extend beyond the order *Picornavirales*. We propose to name the new motif “I motif” in accordance with the established nomenclature for previously described motifs and homomorphs. A literature search revealed that several FMDV variants with amino acid substitutions in the region of the I motif have been investigated. Pro36 to Lys, Ala37 to Val, and Leu39 to Phe were all non-viable, supporting the hypothesis that this RdRp region is critical for the enzymatic function of the protein (Xie et al., 2014). Interestingly, an Ala38 to Val substitution changed the fidelity of the FMDV RdRp (Zeng et al., 2014). This variant was selected as ribavirin-resistant during exposure to ribavirin and demonstrated a 1.65-fold increase in fidelity compared with the wild type FMDV (Zeng et al., 2014), a finding that is in line with similar reports on other polymerases (Mansky and Cunningham, 2000; Pfeiffer and Kirkegaard, 2003). While no specific function has as yet been assigned to the I motif, its high level of conservation warrants further investigation. Future studies should be directed at its possible involvement in regulating polymerase fidelity.

## Calicivirus RdRp Inhibitors

RNA-dependent RNA polymerases are attractive targets for antiviral intervention, because these enzymes are indispensable for virus replication and are very different from any of the host polymerases, which greatly reduces off target effects. RdRp inhibitors can be classified into two major groups: nucleoside analogs (NAs) and non-nucleoside inhibitors (NNIs) (Table 4). NAs are treated by an RdRp as “normal” nucleotides (once an NA is phosphorylated and is in its active form). When they are incorporated into a nascent RNA strand, they can cause a termination of the RNA synthesis or lethal mutagenesis (Galmarini et al., 2001; Costantini et al., 2012). NNIs are aimed to bind an RdRp allosterically, i.e., they bind outside of the active center (Caillet-Saguy et al., 2011; Netzler et al., 2017).

**Table 4:** Calicivirus RdRp inhibitors.

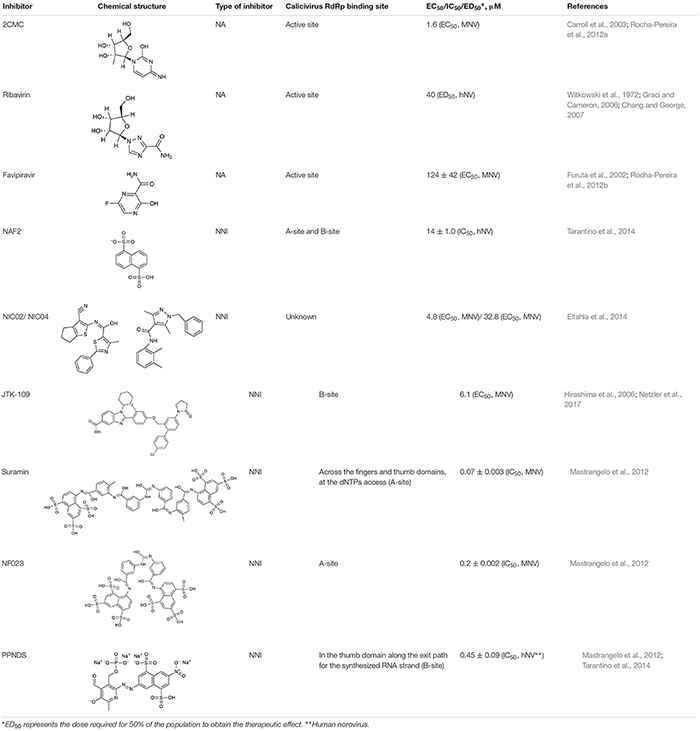

### Nucleoside Analogs

#### 2CMC

The active 5′-triphosphate form of 2′-C-methylcytidine (2CMC) is an HCV polymerase inhibitor that competes with the nucleotide cytidine triphosphate (CTP) for binding to the active site of RdRps. Incorporation of 2CMC into a nascent RNA strand leads to the termination of RNA synthesis. In cell culture, this compound is also active against *Dengue virus*, *Yellow fever virus*, and *West Nile virus* (Pierra et al., 2006). 2CMC also inhibits calicivirus replication in cell culture: as demonstrated by time-of-drug-addition assays, 2CMC inhibited MNV replication and plaque formation (Rocha-Pereira et al., 2012a). Furthermore, 2CMC was able to “cure” cultured cells from Norwalk virus replicons (Rocha-Pereira et al., 2013).

#### Ribavirin

Ribavirin (1-β-D-ribofuranosyl-1,2,4-triazole-3-carboxamide) mimics the guanosine nucleotide and inhibits the replication of a broad range of DNA and RNA viruses (Kanda et al., 2004; Leyssen et al., 2005; Graci and Cameron, 2006). In cell culture experiments, ribavirin significantly reduced norovirus replicon RNA production (Chang and George, 2007). Various mechanisms of the ribavirin-mediated inhibitory effect on virus replication have been proposed, including indirect mechanisms such as guanosine triphosphate (GTP) depletion via the downregulation of inosine monophosphate dehydrogenase, an enzyme that catalyzes GTP synthesis. More direct mechanisms include the ribavirin incorporation into the nascent RNA strand, which may increase mutation frequencies and lead to an “error catastrophe” (Graci and Cameron, 2006).

#### Favipiravir (T-705)

Originally, T-705 (6-fluoro-3-hydroxy-2-pyrazinecarboxamide), a purine nucleoside analog, was developed as an influenza virus inhibitor. T-705 is a prodrug which is turned into its active form (favipiravir-ribofuranosyl-5′-triphosphate) by cellular enzymes (Furuta et al., 2002, 2013). This compound proved also to be a potent inhibitor of bunyaviruses, arenaviruses, and flaviviruses (Gowen et al., 2007; Morrey et al., 2008). Furthermore, it inhibits MNV replication in cell culture, although at a relatively high EC_50_ (half maximal effective concentration) (Rocha-Pereira et al., 2012b). The mechanism through which favipiravir inhibits virus multiplication is most probably lethal mutagenesis, because this NA in its active form is incorporated opposite C and U by “susceptible” RdRps (Jin et al., 2013). This hypothesis was confirmed when increased mutation frequencies were observed in MNV-infected mice after the treatment with favipiravir (Arias et al., 2014). Moreover, after the treatment of a human norovirus-infected patient with favipiravir, a distinct viral variant was observed that differed greatly from all variants that were detected before the treatment commenced and contained 118 non-synonymous substitutions (Ruis et al., 2018).

### Non-nucleoside RdRp Inhibitors

#### Suramin, NF023, and PPNDS

Suramin, NF023, and PPNDS are naphthylurea derivatives. Originally, Suramin was developed as a medication for African sleeping sickness and river blindness (Voogd et al., 1993). However, Suramin and NF023 also inhibit a broad range of viruses, including human norovirus and MNV. The calicivirus RdRps were inhibited in a dose-dependent manner indicating a binding of the drug to the free enzyme or enzyme-substrate complex (Mastrangelo et al., 2012). In norovirus RdRps, there are two defined binding sites for naphthylurea derivatives: one site (retrospectively named “A-site”) is located across the fingers and thumb domains where NTPs access the active site (Mastrangelo et al., 2012), the other one is named the “B-site” and is situated in the thumb domain (Tarantino et al., 2014). The A-site was identified by studying the crystal structures of the MNV RdRp in a complex with Suramin and NF023 (Mastrangelo et al., 2012). The B-site is located along the exit path for the synthesized RNA strand and was identified by analyzing a crystal structure of the RdRp in complex with NAF2 (naphthalene-1,5-disulphonic acid). NAF2 is a fragment derived from the aforementioned compounds (Suramin and NF023) and is likely to represent the most active inhibitory part of larger naphthylurea derivatives. The naphthalene sulfonate-based compound pyridoxal-50-phosphate-6-(20-naphthylazo-60-nitro-40,80-disulfonate) tetrasodium salt (PPNDS) was selected in a docking assay (a method widely used to identify drug binding sites) for further characterization of the B-site. Subsequent studies proved that PPNDS inhibits the RdRp at a much lower IC_50_ (half maximal inhibitory concentration) value compared with NAF2 (Tarantino et al., 2014).

#### NIC02 and NIC04

Phenylthiazole carboxamide (NIC02) and pyrazole acetamide (NIC04) are novel compounds that inhibit GII.4 norovirus RdRps at relatively low IC_50_ values. The mode of inhibition was determined by examining the kinetics of GTP incorporation in the presence of increasing concentrations of these inhibitors. With higher inhibitor concentrations, the reaction velocity and the substrate affinity decreased, suggesting that these compounds can bind both free enzyme and the enzyme-substrate complex (Eltahla et al., 2014). The inhibitory potential of both compounds was also tested using calicivirus RdRps from sapovirus GII, MNV, and different human norovirus strains. NIC02 has a broad inhibitory range (as far as calicivirus RdRps are concerned), which suggests that it binds within a conserved but as yet unidentified motif (Eltahla et al., 2014).

#### JTK-109

JTK-109 is a benzimidazole derivative that is known as an allosteric inhibitor of the HCV RdRp (Hirashima et al., 2006). JTK-109 also possesses inhibitory activity against a variety of caliciviruses (including members of the genera *Norovirus*, *Sapovirus*, and *Lagovirus*), as measured in a quantitative fluorescent *de novo* RdRp activity assay (Eltahla et al., 2013; Netzler et al., 2017). In cell culture experiments, this compound inhibited MNV plaque formation and virus growth (Netzler et al., 2017). Using molecular docking, (Netzler et al., 2017) showed that JTK-109 targets calicivirus RdRps by binding to the B-site of the thumb domain.

## Outlook

Caliciviruses, like almost all other RNA viruses, depend on their RdRps for genome replication. All virus RdRps possess a conserved core structure that is different from cellular polymerases. This dependency on a unique enzyme class provides a soft target for antiviral intervention. However, little information is currently available on how exactly calicivirus RdRps initiate, elongate, and terminate RNA synthesis, how they contribute to the establishment of viral replication factories, and how they escape host cell innate immune responses. Research into the replication of human caliciviruses is especially lacking compared with other small RNA viruses, such as picornaviruses. Norovirus research has long relied on surrogate models or cell-free *in vitro* studies, but recent progress with organoid culture techniques has provided new models that will soon allow the study of RdRp functions in the context of genuine virus replication and host cell countermeasures. As RdRps are often the principal targets in drug-based antiviral therapies, a better understanding of their enzymatic activities and interactions with viral and cellular partners will likely aid in the development of a new generation of highly effective and more specific antivirals.

## Author Contributions

TS and MF developed the conceptual outline. ES, NU, and MF wrote the manuscript (ES wrote the first draft). All authors contributed to editing and revising the manuscript. All authors read and approved the final manuscript.

## Conflict of Interest Statement

The authors declare that the research was conducted in the absence of any commercial or financial relationships that could be construed as a potential conflict of interest.
